# Investitionen und Konsum: wirtschaftspolitische Handlungsoptionen zur Jahresmitte 2020

**DOI:** 10.1007/s10273-020-2675-6

**Published:** 2020-07-06

**Authors:** Michael Hüther

**Affiliations:** Institut der deutschen Wirtschaft Köln e.V., Konrad-Adenauer-Ufer 21, 50668 Köln, Deutschland

**Keywords:** E32, E62, H12

## Abstract

Der historisch einmalige Einbruch der gesamtwirtschaftlichen Leistungen in der Corona-Krise macht wirtschaftspolitische Maßnahmen dringend erforderlich. Wenn die Maßnahmen sich auf die Nachfrageseite richten, müssen sie mit dem richtigen Timing, zielgenau und befristet gestaltet sein. Die Lenkungswirkung sollte dabei in den Hintergrund treten. Ein Maßnahmenkatalog, der von Steuersenkungen bis hin zu Direktzahlungen reicht, ist sinnvoll.

## Abstract (en)

The historically unprecedented slump in overall economic output due to the coronavirus crisis creates an urgency for economic policy responses. Demandside measures must be timely, targeted, and temporary. Addressing policy objectives beyond the economic recovery should not be the focus right now. A package of measures ranging from tax cuts to direct payments is appropriate.

Die Corona-Krise hat einen kräftigen, historisch einmaligen Einbruch der gesamtwirtschaftlichen Leistung ausgelöst. Nach der ersten Schätzung des Statistischen Bundesamts ist das Bruttoinlandsprodukt (BIP) im ersten Vierteljahr 2020 um 2,2 % gegenüber dem Vorquartal geschrumpft, während für das zweite Quartal mit etwa 12 % in laufender Rate eine deutlich stärkere Schrumpfung zu erwarten ist. Alle Prognosen erwarten eine Erholung ab dem dritten Quartal. Dabei wird davon ausgegangen, dass wesentliche Blockaden auf der Angebotsseite weitgehend gelockert oder nicht mehr wirksam sind und dass die Nachfrageseite wieder anläuft.

Infolge weiterhin gestörter Lieferketten und Einschränkungen beim Einsatz der Beschäftigten dürften die Produktionsbetriebe noch länger – bis Jahresende – unter Produktivitätsverlusten leiden. Schwerwiegender dürften die Nachfrageprobleme im weiteren Jahresverlauf wirken. Hier fehlt zunächst die Nachfrage aus vielen Auslandsmärkten. Dazu dürften im zweiten Halbjahr 2020 Belastungen bei der Binnennachfrage zum Tragen kommen, wenn möglicherweise nach der Sommerpause das Ausmaß der Arbeitsplatzverluste greifbar wird und dadurch das Beschäftigungsrisiko allgemein spürbar deutlich ansteigt. Zudem erleiden nicht wenige private Haushalte durch Kurzarbeitergeld, aber auch die Einschränkungen beim Arbeitseinkommen eine Minderung der verfügbaren Einkommen.

**Prof. Dr. Michael Hüther** ist Direktor des Instituts der deutschen Wirtschaft (IW) in Köln und Hochschullehrer an der European Business School in Oestrich-Winkel.

## Voraussetzungen für eine Nachfragepolitik

Wenn sich die Produktion auf Basis dieser Maßnahmen stabilisiert und wieder leicht anläuft, dann kann das zusammen mit anderen Stimmungsaufhellern (wie beispielsweise Urlaubsreisen ins Ausland) ein Umfeld begründen, in dem nachfragepolitische Instrumente sinnvoll sein können. Man läuft mit Nachfragepolitik nicht mehr Gefahr zu versuchen, mit einer offenen Hand ein fallendes Messer aufzuhalten. Die Lockerung der angebotspolitischen Restriktionen öffnet grundsätzlich den Raum für eine wirksame nachfragepolitische Intervention. Notwendig wird diese, wenn eine volkswirtschaftlich bedeutsame Unterauslastung der heimischen Kapazitäten registriert wird und wenn dies auf einer Einkommensbeschränkung der privaten Haushalte beruht – entweder durch tatsächliche Einkommensverluste (Kurzarbeit und Arbeitslosigkeit) und den deshalb als notwendig erachteten Neuaufbau von Ersparnis oder durch erwartete/befürchtete Einkommensverluste wegen sich abzeichnender, drohender Arbeitslosigkeit.

Nachfragepolitik muss „timely, targeted, temporary" sein und sollte keine Lenkungswirkung haben, jedenfalls nicht als vorrangiges Ziel (Bardt et al., 2020). Soweit damit zugleich Modernisierungsfortschritte verbunden werden können, ohne die konjunkturpolitische Reaktion zu schwächen, sollen diese Potenziale genutzt werden. In der Realität geht es vermutlich eher darum, negative Nebenwirkungen (z. B. hinsichtlich des Klimaschutzes) möglichst klein zu halten. Dahinter steht die Tinbergen-Überlegung, dass jedes Ziel durch ein Instrument verfolgt wird und eine Verwässerung des Instrumenteneinsatzes durch

Abbildung 1
**Fiskalpolitische Antworten auf die COVID-19-Pandemie**
in % des nationalen Bruttoinlandsprodukts

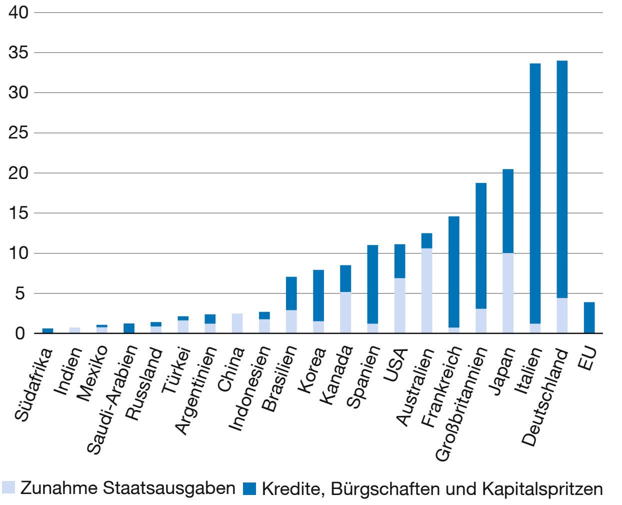

Quelle: Internationaler Währungsfonds, Institut der deutschen Wirtschaft.

Abbildung 2
**Aggregierte Gütergruppen, Deutschland 2016**
in % des gesamten Güteraufkommens, Angaben auf Basis von Input-Output-Tabellen

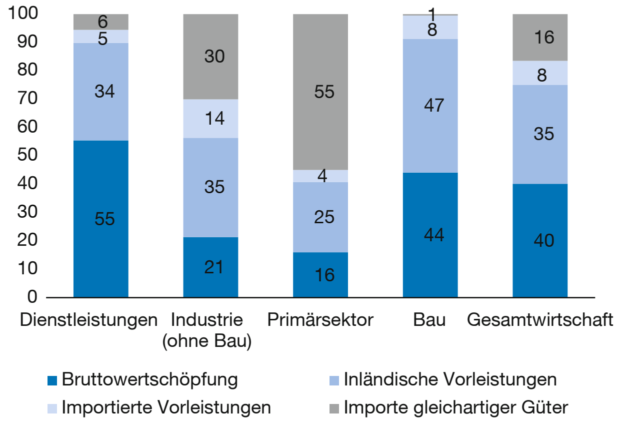

Quellen: Statistisches Bundesamt; Institut der deutschen Wirtschaft.

mehrere Ziele oder Vorgaben zugleich den Wirkungsgrad der Politik verringert, die Kosten in die Höhe treibt und am Ende weder zeitgerecht noch zielgenau ist und damit auch hinsichtlich der Zeitdauer aus dem Rahmen läuft. Wer also nachfragepolitisch kurzfristig und kräftig wirksam handeln will, der muss anders als bei einer wachstumsorientierten Investitionsstrategie auf ergänzende Zielvorgaben weitgehend und primär verzichten.

Eine besondere Herausforderung stellt sich unter den Bedingungen einer Stabilitätsökonomie oder gar einer Deflationsökonomie, weil dann die Konsumenten auch bei nachfragestimulierenden Maßnahmen ihre eigenen finanziellen Mittel schonen, da sie durch Zuwarten keinem Preisrisiko ausgesetzt werden oder noch darauf hoffen können, dass die Preise fallen und sie einen Realeinkommensgewinn erzielen. Hilfreich kann deshalb für die Durchschlagskraft einer Nachfragepolitik sein, wenn zuvor aufgrund von Angebotsproblemen oder aufgrund von preislichen Nachholeffekten im Einzelhandel und bei Dienstleistungen zum Ausgleich des Nachfrageausfalls während des Lockdowns ein transitorischer Inflationsimpuls entsteht.

Kritisch diskutiert werden die Sickerverluste einer Nachfragepolitik durch positive Effekte auf die Importnachfrage. Das relativiert sich mit Blick auf europäische Sickereffekte, da Deutschland durch seine Vernetzung dadurch einen wichtigen Stabilisierungsbeitrag für Europa leisten kann (Kolev und Obst, 2020). Tatsächlich könnte eine europäische Koordinierung nachfragepolitischer Aktivitäten den Wirkungsgrad insgesamt erhöhen. Dies gelang in der globalen Finanzkrise 2009 grundsätzlich gut und fehlt diesmal weitgehend. Immerhin liegen nun verschiedene Vorschläge für eine europäische Aktion vor (Merkel-Macron, EU-Kommission). Die Staaten zeigen in Höhe und Struktur bislang ein sehr unterschiedliches krisenpolitisches Engagement (vgl. Abbildung 1).

Bei aller europäischen Verantwortung wäre es natürlich volkswirtschaftlich vertan, wenn man faktisch jene Sektoren besonders fördert, die eine besonders hohen Importanteil (Fertigwaren und Vorleistungen) aufweisen. Deshalb sollte volkswirtschaftlich wie politisch vor allem der Inlandskonsum – als Konsum von Produkten mit einem hohen inländischen Wertschöpfungsanteil bzw. geringem Importanteil – gefördert werden. Abbildung 2 beziffert auf Basis der Input-Output-Tabellen genau jenen Anteil. Danach bieten Dienstleistungen und Bau den größten Inlandseffekt mit fast 90 %, gefolgt von der Industrie, die einen Inlandsanteil von immerhin 55 % aufweist. Die sektorbezogenen Daten zeigen eine erhebliche Differenzierung, so sind die Importanteile bei pharmazeutischen Produkten (66 %), Datenverarbeitungsgeräten, optischen und elektronischen Geräten (68 %), elektrischen Ausrüstungen (52 %), auch Möbeln (54 %) relativ hoch, bei Kraftwagen (inklusive Teile) mit 36 % eher gering (zwei Drittel davon Vorleistungen).

Es wird deutlich, wie kleinteilig das nachfragepolitische Geschäft wird, wenn man nach differenzierten Förderlogiken Ausschau hält. Hinzu kommt, dass diese Wertschöpfungsanteile noch gespiegelt werden müssen mit dem Wertschöpfungs- und Beschäftigungsanteil der jeweiligen Branchen und der Bedeutung dieser Branche für die Wertschöpfungsketten. Tatsächlich hat sich im Zuge des Lockdowns gezeigt, dass auch in diesem Wirkungszusammenhang die Automobilbranche von besonderer Bedeutung ist und ihrer Rolle als Schlüsselindustrie gerecht wird. Denn im Maschinenbau und in der Chemischen Industrie konnte in den vergangenen Wochen dort, wo es nicht um Vorleistungen für den Automobilbereich ging, noch mit relativ stabiler Auslastung die Produktion gehalten werden.

Im Sinne einer zeitlichen und sachlichen Zielgenauigkeit sowie Wirksamkeit bei zeitlich befristeter nachfragepolitischer Intervention lässt sich eine Reihe bekannter und erprobter Instrumente ebenso anführen wie theoretische Konzepte (vgl. Tabelle 1). Die dreifache Konditionierung – timely, targeted, temporary – soll sicherstellen, dass der konjunkturelle Impuls zeitgerecht und stark zum Tragen kommt. Hinter der Bedingung „targeted" verbergen sich weitere Kriterien einer zielgenauen Wirksamkeit (Brügelmann, 2010): Das relevante Produktionspotenzial ist unterausgelastet (Betroffenheit); die Maßnahmen adressieren Wirtschaftsbereiche mit hoher Netzwerkintegration durch Wertschöpfungsverbünde und Lieferketten (Hebeleffekte); eine Verdrängung von Nachfrage an anderer Stelle ist nicht plausibel und ebenso führen konsumstützende Maßnahmen nicht lediglich zu steigender Ersparnis (Zusätzlichkeit). Schließlich kämpft man gegen Windmühlen, wenn Faktoren das allgemeine Vertrauen der Konsumenten belasten, die nicht nachfragepolitisch direkt zu adressieren sind; die Sorge um den eigenen Arbeitsplatz muss deshalb vor allem von Unterauslastung und Nachfragemangel abhängig sein.

## Vorbild Finanzkrise

Wichtige Hinweise liefern auch die Erfahrungen des Krisenmanagements während der Finanzkrise. Erstens war bemerkenswert, wie schnell es seinerzeit gelang, durch das neue G20-Format der Staats- und Regierungschefs bereits im November 2008 ein gemeinsames Krisenverständnis zu entwickeln, einen Rahmen für die angemessene wirtschaftspolitische Reaktion zu formulieren und damit eine internationale Orchestrierung der Krisenpolitik zu erreichen. Da alle größeren Volkswirtschaften in entsprechender Weise handelten, waren Sickereffekte über Importe kein Argument gegen die Wirksamkeit der Nachfragepolitik. Zweitens wurde damals sehr umfangreich gehandelt, bereits im Herbst 2008 mit dem Konjunkturpaket I und im Frühjahr 2009 mit dem Konjunkturpaket II. Es zeigt sich, dass diese Pakete ein Investitionsprogramm mit befristet ausgestalteten konjunkturellen Impulsen zusammenbrachten; insgesamt verband sich damit ein Finanzvolumen von 104 Mrd. Euro (Leibniz-Institut für Wirtschaftsforschung Halle [IWH], 2015).

Neue Wege ist man mit dem einmaligen Kindergeldzuschuss und der Umweltprämie gegangen. Einerseits sollten Haushalte mit hoher Konsumquote zusätzliche Spielräume für Ausgaben erhalten, andererseits sollte über die Schlüsselbranche Automobil ein besonderer Hebel genutzt werden. Dabei wurden ökologische Aspekte insofern berücksichtigt, als die Umweltprämie von Fahrzeughaltern für ein mindestens neun Jahre altes Altfahrzeug, das für mindestens ein Jahr auf den Halter zugelassen war, beantragt werden konnte, wenn dieses verschrottet und gleichzeitig ein umweltfreundlicher Neu- oder Jahreswagen ab Abgasnorm Euro 4 gekauft und zugelassen wurde. Die Prämie betrug 2.500 Euro und wurde für Zulassungen bis zum 31. Dezember 2009 gewährt, das Gesamtvolumen von 5 Mrd. Euro war am 2. September 2009 ausgeschöpft.

Die Wirksamkeit der Konjunkturpakete kann einerseits daran gemessen werden, dass international koordiniert und rechtzeitig gehandelt wurde, sodass positive Erwartungseffekte plausibel waren, jedenfalls die traditionelle Kritik an nachfragepolitischen Maßnahmen in den Hintergrund trat (IWH, 2015). Andererseits spricht die Ausgestaltung und vor allem die Befristung dafür, dass sich bei den Instrumenten eine schnelle Wirkung – wenn auch mitunter vor allem Vorzieheffekte – einstellte. Insgesamt war die Kritik mit Blick auf die übliche Verzögerung durchaus berechtigt, wenngleich nachfolgende Überhitzungserscheinungen nicht zu diagnostizieren waren.[1] Auch auf Basis dynamisch stochastischer gesamtwirtschaftlicher Gleichgewichtsmodelle ergibt sich ein insgesamt durchaus positives Urteil über die fiskalpolitischen Pakete der Jahre 2008 und 2009.[2]

1 „Im Jahr 2009 wurde zwar ein gewisser konjunktureller Effekt erreicht; der initiale Multiplikator der Maßnahmen insgesamt dürfte zwischen 1,3 und 1,5 gelegen haben; maßgeblichen Anteil daran hatten die Umweltprämie und das Bürgschaftsprogramm Wirtschaftsfonds Deutschland. Aber die Maßnahmenpakete entfalteten erst während des Aufschwungs der Jahre 2010 und 2011 ihre volle Wirkung. Es gibt Hinweise darauf, dass die Erwartung der stimulierenden Effekte der expansiven Finanzpolitik in den Folgejahren auch zur Stabilisierung des Vertrauens der privaten Haushalte und Unternehmen beigetragen und die gesamtwirtschaftliche Nachfrage bereits im Jahr 2009 gestützt hat. Dieser Effekt lässt sich jedoch kaum quantifizieren." (IWH, 2015, 103).

2 „Our estimates hint at the overall positive effects of fiscal policy on German output in the years 2009 and 2010, most of which can be attributed to government consumption, investment, transfers and changes in labor tax rates including social security contributions" (Drygalla et al., 2017).

Tabelle 1
**Ausgewählte Nachfrageimpulse und Kriterien der Wirksamkeit**

| Instrument | zeitgerecht | sachgerecht | | | | befristet |
|---|---|---|---|---|---|---|
| | | Privater Konsum | Sektor-Betroffenheit | Hebeleffekt | Zusätzlichkeit | |
| Mehrwertsteuer-Senkung (allgemeiner Satz) | Ja | Verteilungspolitisch ja, nur privater Konsum | Nicht spezifisch auf unterausgelastete Potenziale, aber Breitenwirkung | Sickerverluste unvermeidbar | Unsicher | Ja |
| Konsumgutscheine | Ja | Nur privater Konsum | Begrenzt auf unterausgelastete Potenziale fokussierbar | Sickerverluste allenfalls regional eingrenzbar | Faktisch „transfer in kind“ (sicherer), gegebenenfalls Vorzieheffekte | Ja |
| Kaufprämie | Ja | Nur privater Konsum | Auf unterausgelastete Potenziale fokussierbar | Hebel, wenn Sickerverlust branchenbezogen eingrenzbar | Faktisch „transfer in kind“ (sicherer), gegebenenfalls Vorzieheffekte | Ja |
| Helikopter-Schwundgeld | Ja | Nur privater Konsum | Nicht spezifisch auf unterausgelastete Potenziale | Gesamtwirtschaftlich ja, Sickerverluste unvermeidbar | Unsicher | Ja |
| Absenkung der Umlage nach dem Erneuerbare-Energien-Gesetz (EEG) | Unsicher | Verteilungspolitisch ja, heterogen unter Unternehmen | Nicht spezifisch auf unterausgelastete Potenziale | Sickerverluste unvermeidbar | Unsicher | Ja |
| Kürzung der Sozialbeiträge | Unsicher | Verteilungspolitisch (wegen Konsumquote) ja | Nicht spezifisch auf unterausgelastete Potenziale | Sickerverluste unvermeidbar | Unsicher | Ja |
| Vorziehen der Soli-Abschaffung | Nein | Unsicher | Nicht spezifisch auf unterausgelastete Potenziale | Sickerverluste unvermeidbar | Unsicher, Erwartungseffekt höherer Nettoeinkommen | Ja (Vorzieheffekt ist temporär) |
| Einkommensteuer Grundfreibetrag & Eingangssteuersatz | Nein | Verteilungspolitisch (wegen Konsumquote) ja | Nicht spezifisch auf unterausgelastete Potenziale | Sickerverluste unvermeidbar | Plausibel, da Haushalte mit höherer Konsumquote | Nein |
| Einmaliger Kindergeldzuschuss | Unsicher | Verteilungspolitisch (wegen Konsumquote) ja | Nicht spezifisch auf unterausgelastete Potenziale | Sickerverluste unvermeidbar | Plausibel, da Haushalte mit höherer Konsumquote | Ja |

Quelle: eigene Zusammenstellung.

## Nachfragepolitische Empfehlungen

Angesichts der skizzierten Erfahrungen und der kategorialen Einordnung nachfragepolitischer Instrumente lassen sich die folgenden Hinweise ableiten:

### Mehrwertsteuer senken

Naheliegend erscheint bei gesamtwirtschaftlicher Unterauslastung die Idee einer *befristeten Minderung der Mehrwertsteuer* (nur allgemeiner Satz, sodass Grundkonsum ausgenommen würde). Wegen der stark regressiven Wirkung der Mehrwertsteuer dürfte hier der Konsumeffekt besonders groß sein; im untersten Einkommensdezil belastet die Mehrwertsteuer das Bruttoeinkommen drei Mal so stark wie im obersten Dezil; im zweiten Dezil schlägt sie immerhin noch mit dem Doppelten zu Buche als im zweitobersten, neunten Dezil (Beznoska, 2020). Dabei ist sicherzustellen, dass die Steuerreduktion an die Konsumenten weitergegeben wird, um den gewünschten Impuls bei der Konsumnachfrage auszulösen; dabei stehen möglicherweise Menükosten im Wege, die aber digital zu mindern sind. Grundsätzlich bleibt das Problem, dass die Preise bei einer Senkung der Mehrwertsteuer eher nur unvollständig angepasst werden, tendenziell wird eher bei Unternehmen mit höherer Gewinnmarge die Senkung an Konsumenten weitergereicht (Benzarti et al., 2020). Die Abwicklung wäre sofort möglich, d. h. man kann den Kaufpreis entsprechend mindern, der Einzelhändler muss demgemäß nur geringere Mehrwertsteuer abführen. Für eine verlässliche Entlastung der Konsumenten wäre zu prüfen, ob nicht ein befristeter Vorsteuerabzug der Mehrwertsteuer bei der Einkommensteuer oder eine direkte Erstattung der gezahlten allgemeinen Mehrwertsteuer in einem Zeitraum beim Finanzamt beantragt werden kann.

### Konsumgutscheine

In Form eines allgemeinen Gutscheins oder als konsumbezogene Prämie wird über entsprechende Vorschläge diskutiert. Man kann auf diese Weise die privaten Haushalte gut erreichen und direkt den Konsum adressieren, freilich können die geschonten Eigenmittel in die Ersparnis gehen. Wenn man über Kaufprämien oder Konsumgutscheine nachdenkt, dann ist der gesamtwirtschaftliche Hebeleffekt zu berücksichtigen, falls Sektoren adressiert werden, die eine hohe Unterauslastung mit krisenbedingt hohen Lagerbeständen verbinden. Hier kann durch das Auflösen der Nachfrageverstopfung ein größerer Effekt erzielt werden.

- *Umweltprämie*: Dafür wird auf die Hebelwirkung der Prämie aus dem Jahr 2009 verwiesen. Tatsächlich stiegen damals die Autokäufe an, allerdings auch zulasten anderer Konsumsegmente. So nahmen im Jahr 2009 die Ausgaben privater Haushalte für den Kauf von Kfz um 20,5 % zu, der private Konsum ohne Kraftfahrzeuge ging indes um 0,5 % zurück; insgesamt führte dies zu einer Zunahme des privaten Konsums um 0,4 % (Statistisches Bundesamt, 2010). Das ist freilich kein Gegenargument, denn diese Konsumverlagerung hat den volkswirtschaftlichen Hebeleffekt erhöht. Viele Kunden haben damals den Autokauf vorgezogen, sodass im Folgejahr die entsprechende Nachfrage fehlte. Der Marktanteil ausländischer Autobauer stieg sprunghaft um fast elf Prozentpunkte auf rund 55 %; das ist angesichts einer Importquote in dieser Branche von 36 % einerseits nicht überraschend, eröffnet aber andererseits einen Blick auf die europäische Bedeutung der Automobilbranche; ein Problem ist dies nur bei nationaler Engführung. Insgesamt zeigt sich im Rückblick ein positives konjunkturelles Bild (IWH, 2015).

Diesmal existiert bereits eine Umweltprämie, nämlich für Autos mit Batterie- und Brennstoffzellenantrieb (6.000 Euro) und Hybridelektrofahrzeug (4.500 Euro) bis 40.000 Euro Nettopreisliste (bis 65.000 Euro beträgt die Prämie 5.000 Euro). In diesen Segmenten bestehen auch derzeit keine Kapazitätsprobleme. Denkbar wäre eine Ausweitung der Prämie auf Dieselantrieb (Euronorm 6d) sowie moderne Otto-Motoren (3.000 Euro) bis zu einem Nettolistenpreis von 65.000 Euro, sodass sich der $CO_2$-Ausstoß im Flottendurchschnitt mindern würde.

- *Digitales Helikoptergeld als Schwundgeld*: Helikoptergeld hat ursprünglich eine andere Funktion: Es soll Zentralbankgeld unter das Volk bringen und damit für einen Inflationsimpuls sorgen. Man könnte fiskalische (und nicht geldpolitische) Konsumgutscheine als Schwundgeld ausgestalten, sodass eine mit Zeitablauf in Stufen sinkende Attraktivität zu erheblichen Vorzieheffekten führen dürfte. Sickereffekte durch Importstimulierung sind nicht zu vermeiden. Damit diese Gutscheine zusätzlich wirken, könnte man den anteiligen Einsatz eigener Mittel fordern, sodass die Ersparnis nicht begünstigt wird.

### Entlastung der unteren und mittleren Einkommen

Entlastung der unteren und mittleren Einkommen, die traditionell eine höhere Konsumquote aufweisen und gegenwärtig möglicherweise besonders von Einkommenssorgen und -verlusten geprägt sind.

- *Befristete Absenkung der Umlage nach dem Erneuerbare-Energien-Gesetz (EEG)*: Diese Maßnahme hat den Charme, dass sie vor allem bei Haushalten mit kleineren und mittleren Einkommen wirkt, wo die Konsumquote groß ist (vgl. auch Umweltbundesamt, 2020). Um zeitnah Liquiditätseffekte zu realisieren, müsste man Erstattungen über die Stromversorger organisieren. Interessant ist dieser Vorschlag, weil die EEG-Umlage zum 1. Januar 2020 wieder angestiegen ist (auf 6,756 Cent pro kWh) und im unteren (zweiten) Einkommensdezil das Bruttoeinkommen vier (drei) Mal so stark belastet wie im zweitobersten (neunten) Dezil (Beznoska, 2020).

- *Befristete Absenkung der Sozialbeiträge*: Mit diesem Instrument trifft man ebenfalls zielgenauer breitere Einkommensbereiche, die Erhöhung des verfügbaren Einkommens wäre monatlich sichtbar. Der Bundeshaushalt müsste entsprechend einen Zuschuss an die Bundesagentur für Arbeit und an den Gesundheitsfonds vorsehen. Beamte und Selbständige blieben außen vor, was man mit der geringeren Betroffenheit bzw. den gezielten Unterstützungen begründen kann.

- *Einmaliger Kindergeldzuschuss*: Dieses Instrument ist im Konjunkturpaket II genutzt worden. Dafür sprechen in der gegenwärtigen Lage zusätzlich die besonderen Belastungen, denen Familien mit Kindern durch die Einschränkung der Kinderbetreuungseinrichtungen und der Schulen ausgesetzt sind. Die Konsumquote dürfte hier hoch sein, die zeitgerechte Wirkung ist zwar unsicher, aber dennoch erwartbar. Eine Hebelwirkung ist wegen Breitenwirkung und Zusätzlichkeit nicht unplausibel, grundsätzlich ist die konjunkturelle (kurzfristige) Wirkung so kalibrierter Transfers größer als die einer allgemeinen Senkung der Sozialbeiträge (Gechert et al., 2020). Zudem sollte die höhere Bedarfsgerechtigkeit dieses Kindergeldzuschusses – im Vergleich zu einer allgemeinen Transfererhöhung – einen größeren Multiplikator aufweisen (Bayer et al., 2020).

### Einkommensteuersenkung

Eine allgemeine Senkung der Einkommensteuer ist angesichts der insgesamt hohen Steuerquote – diese lag mit

24 % im Jahr 2019 deutlich über dem langjährigen Durchschnitt von 22,7 % – grundsätzlich erwägenswert und für die langfristige Stärkung des Wachstumspotenzials ergänzend zu gezielten Investitionsanreizen und -programmen geboten (Hüther, 2020). Kurzfristig und konjunkturpolitisch ist davon wenig zu erhoffen, die Erwartungseffekte dürften kaum die negative Konsumstimmung drehen.

- *Senkung des Eingangssteuersatzes und Erhöhung des Grundfreibetrags*: Eine gezielte Entlastung der unteren Einkommen ist noch am ehesten zu bedenken. Allerdings führen diese Änderungen dazu, dass der Einkommensteuertarif insgesamt immer weniger anreizkompatibel wird, da die zunehmende Stauchung im Tarifverlauf den Progressionsgrad erhöht.

- *Steuergutschrift*: Als Beispiel dienen hier die USA mit dem Instrument der Steuergutschriften sowie aktuell der Steuererstattungschecks. Denkbar wäre eine Einkommensteuersenkung (dauerhaft, aber über das Vorziehen des Soli temporär wirksam) in Kombination mit einmaliger Auszahlung (Mindeststeuergutschrift 300 oder 500 Euro pro Person), die nicht zurückgezahlt werden muss, wenn die Steuerersparnis eigentlich weniger wäre. Das erreichte auch Haushalte, die wenig Einkommensteuer zahlen, aber eine hohe Konsumquote haben.

- *Abschaffung des Solidaritätszuschlags vorziehen*: Es bleibt mit Blick auf die private Konsumnachfrage nachrangig und wenig zielgenau. Das hilft allerdings (bei kompletter Abschaffung) der Investitionsnachfrage, denn es entlastet Unternehmen von einem faktisch zur Unternehmensteuer mutierten Rest-Soli.

Für die Gestaltung eines konjunkturellen Gesamtpakets ist grundsätzlich zu beachten, dass der Wirkungsgrad mit zunehmender internationaler Koordinierung steigt. Das gilt für die G20, die ein wichtiges Signal senden können, wenn es wie 2008/2009 gelänge, eine Orchestrierung der Krisenpolitik zu organisieren. Das eröffnet grundsätzlich die Möglichkeit, die Hebelwirkung der nationalen Programme zu erhöhen, und könnte bei gemeinsamen Aktionen für spezifische Sektoren eine besondere Wirkung entfalten. Das gilt natürlich direkt für die Europäische Union, in der über einen European Recovery Fund Zuschüsse und Kredite in den Mitgliedstaaten für expansive Maßnahmen zur Krisenremedur verfügbar werden sollen; die Verhandlungen dazu sollten vor der Sommerpause abgeschlossen werden. Dazu gehört auch eine Koordinierung der Anstrengungen für den nicht unplausiblen Fall, dass die in der zweiten Jahreshälfte vermehrt eintretenden Unternehmensinsolvenzen und der Ausfall von Krediten auf die Banken durchschlagen. Dafür stehen der Europäische Stabilitätsmechanismus (ESM) und der Single Resolution Fund (SRF) zur Verfügung und müssen handlungsfähig sein.

## Ein deutsches Konjunkturprogramm

Empfehlenswert für ein deutsches Konjunkturprogramm erscheinen vor dem Hintergrund der dargelegten Befunde und Argumente die folgenden Maßnahmen, bei denen eine Stabilisierung der Unternehmen weiterhin Priorität hat, um Angebotsprobleme zu vermeiden oder zu beseitigen und der Nachfragepolitik den Boden hoher Wirksamkeit zu bereiten. Steuer- und Abgabenerhöhungen – sei es nur als Ankündigung für künftige Zeiten – wären freilich Gift für die Wirksamkeit aller Teile des Programms. Ebenso darf es nicht dazu kommen, dass die expansiven Maßnahmen des Bundes und der Länder durch massive Haushaltskürzungen bei den Kommunen – wo ohnehin der Schwerpunkt der öffentlichen Investitionen verantwortet wird – konterkariert werden. Der Vorschlag des Bundesfinanzministers, dass Bund und Länder den Kommunen in diesem Jahr den Ausfall der Gewerbesteuereinnahmen jeweils hälftig ausgleichen, ist deshalb zu begrüßen.

1. Aufsetzen eines Investitionsprogramms (Dullien et al., 2020) mit den folgenden Elementen:

a. Unter anderem: energetische Gebäudesanierung, Abwrackprämie für Ölheizungen sowie Wärmepumpenprogramm, F&E-Förderung für Schlüsseltechnologien, Altschuldenhilfe für die Kommunen, Infrastrukturgesellschaft für Elektro- und H2-Mobilität, diverse Investitionen in das Humanpotenzial; b. Anpassung der Abschreibungsregeln (degressive Afa, Sonder-Afa) und weitere steuerliche Maßnahmen im Unternehmensteuerrecht (Wissenschaftlicher Beirat, 2020): Verlustrücktrag/Negativsteuer, steuerliche Abzugsfähigkeit der Tilgung für Corona-Kredite, Aussetzung der Mindestbesteuerung, einmalige steuerfreie Rücklage für 2019;

2. Gezielt befristet private Haushalte entlasten durch:

a. Absenkung der EEG-Umlage bis Jahresende um 50 %. Bei einem Planaufkommen von 24 Mrd. Euro und einem Anteil von einem Drittel, der von den privaten Haushalten gezahlt wird, ergäben sich fiskalische Kosten von rund 4 Mrd. Euro.

b. Zuschuss im Rahmen des Kindergelds nach Kinderzahl: 300 Euro für jedes Kind, das im elterlichen Haushalt wohnt, sonstige Regelung wie 2009 im Konjunkturpaket II. Das finanzielle Volumen dürfte bei gut 3,5 Mrd. Euro liegen.

3. Gezielte Kaufimpulse mit hoher gesamtwirtschaftlicher Hebelwirkung:

a. Befristete Minderung der Mehrwertsteuer (allgemeiner Satz, gegebenenfalls Erstattung als Vorsteuer beim Finanzamt). Die Absenkung des allgemeinen Mehrwertsteuer-Satzes um einen Prozentpunkt verursacht Steuerausfälle von rund 1 Mrd. Euro im Monat. Würde man für drei Monate den Satz um 4 Punkte absenken, wären das 12,4 Mrd. Euro, für vier Monate 16,5 Mrd. Euro.

b. Im Zusammenspiel mit den genannten Maßnahmen kann eine Umweltprämie (z. B. in Höhe von 3.000 Euro) für alle Antriebsarten geprüft werden; auch Diesel- (Euronorm 6d) sowie moderne Otto-Motoren (bis 65.000 Nettolistenpreis), sodass sich der $CO_2$-Ausstoß im Flottendurchschnitt mindern würde: Volumen 5,0 Mrd. Euro bis Jahresende 2020 im Windhund-Verfahren verfügbar; Regelung zur Verschrottung ähnlich wie vor zehn Jahren. Steigende Importe werden als Ausdruck europäischer Integration gewertet; die Prämie sollte zu Wirksamkeitssteigerung aber möglichst in den europäischen Nachbarländern – wie in Frankreich bereits beschlossen – gespiegelt werden.

Konjunkturpolitik, daran ist zum Schluss nochmals zu erinnern, muss zuerst und vor allem unter den Gesichtspunkten der zeitlichen und sachlichen Passgenauigkeit sowie der Befristung gesehen werden, was „Strohfeuer“ und „Sickerverluste“ bewusst hinnimmt. Das gehört zum Wesen der Nachfragepolitik. Es verlangt die Bereitschaft, strukturelle Aspekte dabei erst in zweiter Linie berücksichtigen zu können. Zugleich ist langfristig zu denken, um das Wachstumspotenzial zu stärken und die Finanzierung der öffentlichen Haushalte wieder auf eine nachhaltige Basis zu stellen. Das von den Regierungsparteien am 3. Juni 2020 beschlossene Maßnahmenpaket zur Konjunktur- und Krisenbewältigung, Zukunftssicherung und internationalen Verantwortung (BMF, 2020) folgt weitgehend den hier skizzierten Kriterien und Vorschlägen.“

**Title:** ***Investment and Consumption: Economic Policy Options in Mid-2020***

**JEL Classification:** *E32, E62, H12*
